# High-Speed X-Ray Imager ‘Hayaka’ and Its Application for Quick Imaging XAFS and *in Coquendo* 4DCT Observation

**DOI:** 10.3390/s26020434

**Published:** 2026-01-09

**Authors:** Akio Yoneyama, Midori Yasuda, Wataru Yashiro, Hiroyuki Setoyama, Satoshi Takeya, Masahide Kawamoto

**Affiliations:** 1 SAGA Light Source, 8-7 Yayoigaoka, Saga 841-0005, Japan; setoyama@saga-ls.jp (H.S.); kawamoto@saga-ls.jp (M.K.); 2 Department of Health and Nutrition Sciences, Nishikyushu University, 4490-9 Kanzaki, Saga 842-8585, Japan; midori@nisikyu-u.ac.jp; 3 International Center for Synchrotron Radiation Innovation Smart (SRIS), Tohoku University, Katahira 2-1-1, Aoba-ku, Sendai 980-8577, Japan; wataru.yashiro.a2@tohoku.ac.jp; 4 Institute of Multidisciplinary Research for Advanced Materials (IMRAM), Tohoku University, Katahira 2-1-1, Aoba-ku, Sendai 980-8577, Japan; 5 Department of Finemechanics, Graduate School of Engineering, Tohoku University, Katahira 2-1-1, Aoba-ku, Sendai 980-8577, Japan; 6 Graduate School of Dentistry, Tohoku University, Katahira 2-1-1, Aoba-ku, Sendai 980-8577, Japan; 7 Energy Process Research Institute (EPRI), National Institute of Advanced Industrial Science and Technology (AIST), Tsukuba West, 16-1, Onogawa, Tsukuba 305-8569, Japan; s.takeya@aist.go.jp

**Keywords:** 4DCT, time-resolved CT, XAFS, synchrotron radiation, boiling process

## Abstract

**Highlights:**

**What are the main findings?**

- Developed a lens-coupled high-speed X-ray camera, “Hayaka,” capable of high-resolution imaging with a 1 μs minimum exposure time and a 5000 fps maximum frame rate.
- Demonstrated high-speed energy scanning for QXAFS near the Cu K-edge in 0.5 s and achieved in situ 4D-CT observation of the somen noodle boiling process with a 0.5 s time resolution.

**What are the implications of the main findings?**

- The “Hayaka” camera system enables the observation of ultra-fast structural changes and chemical state transitions.
- This high-speed 4D-CT technique opens new possibilities for real-time, three-dimensional analysis of complex dynamic processes in food science and materials engineering.

**Abstract:**

A lens-coupled high-speed X-ray camera, “Hayaka”, was developed for quick imaging of X-ray absorption fine structure (XAFS) and time-resolved high-speed computed tomography (CT) using synchrotron radiation (SR). This camera is a lens-coupled type, composed of a scintillator, an imaging lens system, and a high-speed visible light sCMOS, capable of imaging with a minimum exposure time of 1 μs and a maximum frame rate of 5000 frames/s (fps). A feasibility study using white and monochromatic SR at the beamline BL07 of the SAGA Light Source showed that fine X-ray images with a spatial resolution of 77 μm can be captured with an exposure time of 10 μs. Furthermore, quick imaging XAFS, combined with high-speed energy scanning of a small Ge double crystal monochromator of the same beamline, enabled spectral image data to be acquired near the Cu K-edge in a minimum of 0.5 s. Additionally, an *in coquendo* 4DCT (time-resolved 3D observation of cooking processes) observation combined with a high-speed rotation table revealed the boiling process of Japanese somen noodles over 150 s with a time resolution of 0.5 s.

## 1. Introduction

The brilliance of synchrotron radiation (SR) is four or more orders of magnitude higher than that of X-rays emitted from conventional X-ray tubes. This high-brilliance X-ray allows us to perform various observations with high spatial, temporal, and density resolutions, surpassing conventional X-ray imaging [1]. High temporal-resolution imaging techniques, such as time-resolved absorption spectral imaging (quick imaging X-ray absorption fine structure (XAFS)), have been developed to visualize temporal maps of changes in chemical states and have advanced significantly in many fields, including materials science. In a conventional configuration, a high-speed energy-scanning monochromator is synchronized with a fast X-ray imager that directly records the transmitted intensity, allowing acquisition of time-resolved spectral images during dynamic processes [2,3,4]. This approach has enabled a broad range of operando measurements on Li-ion battery (LIB) electrodes and related materials [2,5,6] and has also been extended to fuel cells [7] and heterogeneous catalysts [8,9]. Furthermore, by integrating with full-field microscopy [10,11,12,13], micron-scale spatially resolved mapping of local chemical states in complex, heterogeneous systems has become feasible, as demonstrated for cathode/anode layers of all-solid-state LIB [14] and Cr-doped dendritic FeOx particles [15].

In parallel with these developments, time-resolved high-speed computed tomography (CT), often referred to as four-dimensional CT (4DCT; three spatial dimensions + time), has emerged as a powerful modality for capturing three-dimensional structural dynamics [16]. By synchronizing a high-speed X-ray imager with rapid sample rotation, high-speed CT enables continuous visualization of evolving internal structures with temporal resolutions down to a few milliseconds’ regime, which has driven rapid expansion of applications in many research fields. Using such 4DCT methodologies, a wide variety of dynamic phenomena have been revealed, including fluid invasion events such as Haines jumps in porous media [17], capillary transport in microfluidic channels [18], carbonation reactions in cementitious materials [19], bubble nucleation and growth in metallic systems [20,21,22], oil penetration in fried foods [23], nucleation and evolution of internal voids in polymer rubbers under tensile loading [24], and a variety of in vivo biomedical processes [25,26]. To mitigate degradation of spatial resolution, temporal resolution, and signal-to-noise ratio at such elevated acquisition speeds, a high-sensitivity phase-contrast X-ray imaging method [27], such as the propagation-based phase contrast method [28] and X-ray grating (Talbot) interferometry [29,30], has been employed to enhance contrast for weakly absorbing or low-Z materials in these observations. In addition, various reconstruction algorithms have been developed to cope with limited-angle and noisy data, such as iterative reconstruction methods [31,32,33] and curved-trajectory back-projection methods [34]. Recently, to address the effects of centrifugal force from high-speed rotation, a multi-beam optical system has been developed that simultaneously irradiates multiple X-rays from different directions to obtain a CT dataset in a single shot. Three-dimensional images of 50 μm-diameter tungsten wire were successfully obtained with a temporal resolution (exposure time) of 1 ms [35].

SAGA Light Source, located in Tosu, Saga Prefecture, Japan, is an SR facility with a stored electron energy of 1.4 GeV, a maximum stored electron current of 300 mA, a circumference of 75.6 m, and 11 beamlines [36]. A superconducting wiggler [37] has been installed in the beamline BL07, providing SR with a brilliance of 10^14^ [photons/s/mrad^2^/0.1% B.W./mm^2^], over 10,000 times higher than that of conventional tube X-rays around 10 keV [38]. Consequently, quick imaging XAFS and 4DCT are expected to be made possible by combining this highly brilliant SR with a high-speed X-ray imager. We have therefore developed a new lens-coupled high-speed X-ray camera, “Hayaka,” consisting of a scintillator, an imaging lens system, and a high-speed visible light scientific complementary metal-oxide semiconductor (sCMOS). We report the detailed specifications of Hayaka and its evaluation results for spatial resolution and sensitivity using a metal chart. We also present the results of quick imaging XAFS targeting copper (Cu) particles with different oxidation states and the results of a time-resolved three-dimensional observation of the boiling process of Japanese *somen* noodles using *in coquendo* 4DCT.

## 2. High-Speed X-Ray Imager ‘Hayaka’

**Figure 1:**
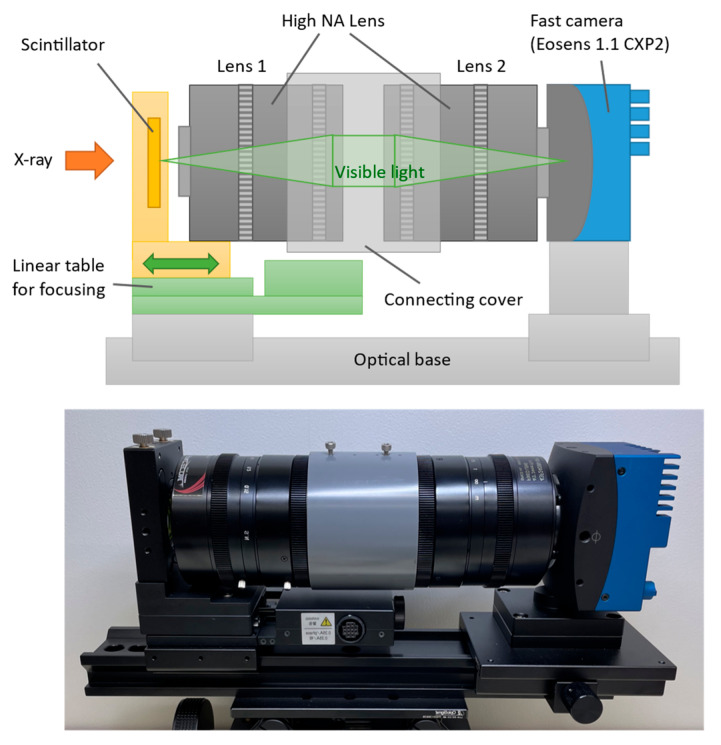
Schematic view (**top**) and photograph (**bottom**) of Hayaka.

Figure 1 shows a schematic view (top) and a photograph (bottom) of the high-speed X-ray camera, Hayaka, which means “very fast” in a Japanese dialect. Hayaka is a lens-coupled type composed of a scintillator, a lens system, a high-speed sCMOS visible light camera, a focus adjustment mechanism, and a base that mounts these components. The scintillator converts incident X-rays into visible light, which is imaged onto the camera’s image sensor surface by the lens system. A LuAG (Lu_3_Al_5_O_12_:(Ce)) crystal plate, which has excellent radiation resistance and highly efficiently converts X-rays into visible light, was used for the scintillator [39]. The plate was 20 mm × 20 mm and was fixed in a commercially available optical lens holder.

The EoSens 1.1CXP2 from Mikrotron GmbH (Unterschleissheim, Germany) was used as the high-speed camera. Its main specifications are shown in Table 1. Its pixel number was 1280 × 864, pixel size was 13.7 μm, and bit resolution was 10 bits (stored as 16 bits). The image transfer rate was 3660 frames per second for a full frame and 5000 frames per second for an 800 × 400 region of interest (ROI) image. The camera interface is CoaXPress 2.0, utilizing 4 channels to transfer image data to the host PC at up to 50 Gbit/s. Consequently, the camera does not have onboard memory. Instead, it rapidly transfers captured image data to the host PC’s memory for temporary storage. After imaging concludes, the host PC saves the image data to a hard disk drive (HDD) or solid-state drive (SSD). Continuous measurement time depends on the installed memory of the host PC. With 64 GB of installed memory, a measurement time corresponding to a 50 GB data capacity can be achieved. For example, if 2000 images with 800 × 400 pixels (0.58 MB) are captured per second, the measurement time will be approximately 40 s.

The lens system employed a back-to-back configuration [40,41] (with lenses facing each other on their backsides) with commercially available high-NA lenses of the specifications listed in Table 2. Although the field of view (FOV) of high-NA lens combinations is constrained by vignetting, these lenses were selected to maximize the optical throughput of the lens system for high-speed X-ray imaging. Five lens combinations (①: No. 1-No. 1, ②: No. 1-No. 2, ③: No. 1-No. 3, ④: No. 3-No. 1, ⑤: No. 3-No. 2) were evaluated in terms of their FOV, brightness, and spatial resolution using white and monochromatic SR at BL07 to select the optimal lens combinations for quick imaging XAFS and 4DCT. Note that since the high-speed camera has a C-mount, only C-mount lenses (No. 1 or 3) can be used for lens 2 (the downstream lens). Since all lenses have the same outer diameter of 98 mm, they were connected by using a polyvinyl chloride (PVC) cylinder, as shown in Figure 1, after setting both the focal length to infinity and the aperture to maximum. A combination of lenses with the same focal length (lens combinations ① and ②) results in a 1:1 magnification, yielding an effective pixel size of 13.7 μm, which is the same as that of the camera sensor.

As shown in Figure 1, the distance between the scintillator and the lens system was adjusted by moving the lens holder that fixes the scintillator by using a linear table driven by a stepping motor. The minimum positioning step of this table is 1 μm (at full step), which is sufficiently smaller than the depth of focus (±40 μm: calculated value). The focal position was adjusted so that the edge of the Au mesh (400 lines and spaces/inch) image, captured using monochromated SR, appeared sharpest.

## 3. Results

### 3.1. Evaluation of Imaging Performance of Hayaka

The imaging performance of Hayaka was evaluated at the beamline BL07 of the SAGA Light Source. The white SR emitted from a superconducting wiggler at BL07 was first shaped into a 10 × 3 mm size with a slit (TC1) installed at the upper stream of BL07. Then, the low-energy component of SR was filtered out with a 0.5 mm-thick water-cooled aluminum filter before the SR irradiated the sample. The peak energy of SR was 20 keV, with a bandwidth of approximately 10 keV [42]. To prevent the spatial resolution from degrading due to diffraction, the sample and the Hayaka scintillator were kept less than 2 mm apart.

**Figure 2:**
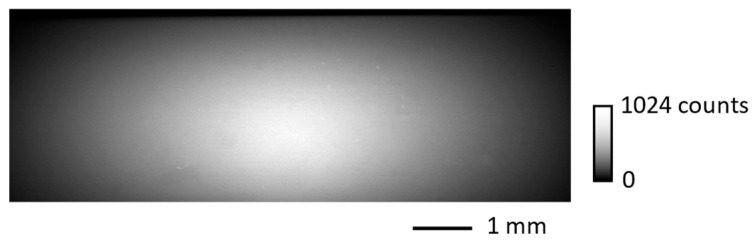
Projection image acquired with an exposure time of 12 μs.

Figure 2 shows the projection image without any sample acquired at an exposure time of 12 μs with a ring current of 200 mA using the lens combination ②. The image size was 8 mm × 3 mm (590 × 220 pixels). The width and height at which the intensity decreases to half its maximum value (the effective width and height) are 5.4 mm (400 pixels) and 2.0 mm (145 pixels), respectively. Since the beam size of the incident white SR was 10 × 3 mm, the horizontal width is limited by the lens’s effective FOV due to vignetting, which closely coincides with the calculated value provided by the lens manufacturer (SPACE), while the vertical height is mostly constrained by the beam size. To overcome these FOV limitations, we plan to reuse lenses [43] with a larger image circle of 40 mm in future work.

**Figure 3:**
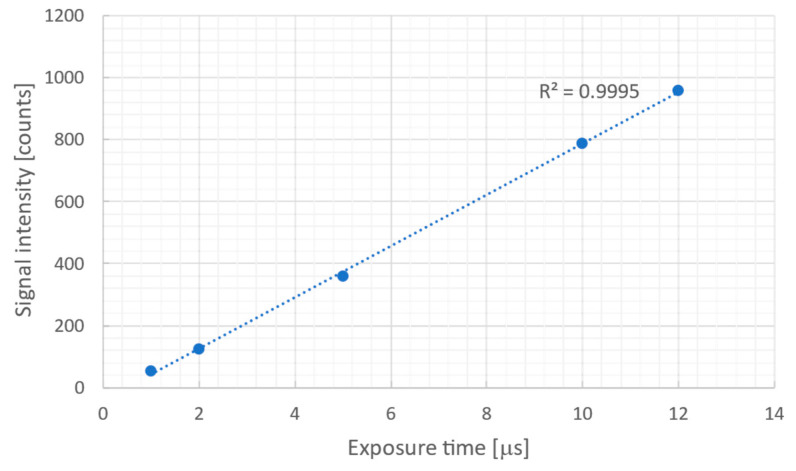
Measured signal intensity at each exposure time. Linearity is ensured up to just before 10-bit (1024 gray levels) saturation.

Figure 3 shows the measured signal intensity (the average of a 10 × 10 pixel area at the center of the projection image) for each exposure time. As can be seen, the signal intensity is proportional to the exposure time, with a correlation coefficient of 0.99, from the camera’s minimum exposure time of 1 μm to 12 μs, just before saturation. Note that this result shows that projection images can be obtained in less than 12 μs exposure times. However, this camera’s maximum frame rate is 5000 fps for an 800 × 400 ROI image. Consequently, the temporal resolution is limited by the frame rate of the camera, resulting in a temporal resolution of 200 μs. Similar measurements were performed for other lens combinations (①, ③–⑤) to evaluate the effective field of view (effective width and height) and relative sensitivity.

**Figure 4:**
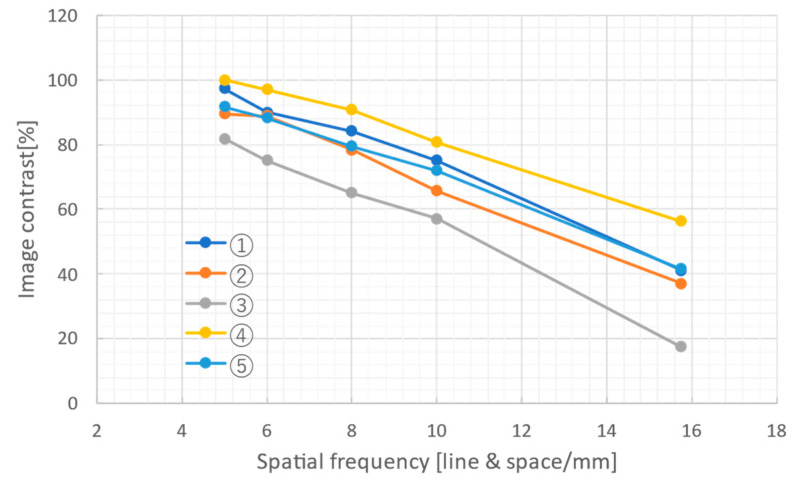
MTF for lens combinations ① to ⑤ calculated from Au mesh and X-ray charts imaged with 10 keV monochromatic SR.

The spatial resolution of each lens combination ① to ⑤ was next evaluated using monochromated SR at BL07. The white SR was shaped into a 5 × 3 mm size by TC1, monochromated to 10 keV by a Ge double crystal monochromator (DCM) [42], and then irradiated onto an Au mesh and an X-ray chart (Moriyama-made, Type 1) for modulation transfer function (MTF). The Au mesh had 200 and 400 lines and spaces (LS)/inch (8 and 16 LS/mm), and the X-ray chart had 10, 8, 6, and 5 LS/mm. The exposure time was set to 40 ms. Figure 4 shows the MTFs (the image contrast at each spatial frequency) calculated from the obtained images by using the method described in our previous paper [39]. These results indicate that lens combination ④ has the highest spatial resolution, maintaining an image contrast of 50% even at 16 LS/mm. Note that spatial resolution in this report is defined by the spatial frequency at which the image contrast attains 50%.

**Figure 5:**
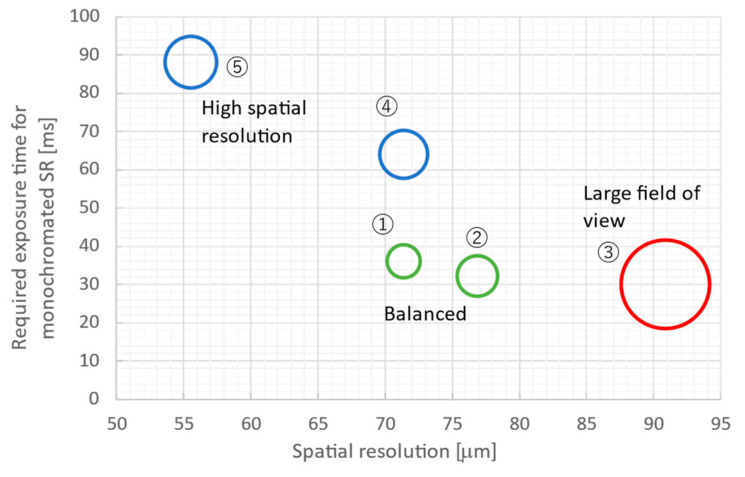
Relationship between spatial resolution, required exposure time for monochromated SR, and observation field of view for lens combinations ① to ⑤. Circle size is proportional to the observed field of view.

Table 3 and Figure 5 summarize and illustrate the evaluation results of the effective width and height, required exposure time, and spatial resolution of each lens combination. Note that the required exposure times are the calculated times needed to obtain a signal intensity of 1000 counts per pixel using white SR filtered by a 0.5 mm-thick aluminum absorber and 10 keV monochromated SR at BL07, respectively. In Figure 5, the size of each circle is proportional to the effective field of view. These results show that lens combinations ① and ② offer a good balance between spatial resolution and exposure time, combination ③ is suitable for wide-field observation, and combinations ④ and ⑤ are suitable for high-resolution observation. For subsequent observations (quick imaging XAFS and *in coquendo* 4DCT), the balanced lens combination (②) was adopted.

### 3.2. Quick Imaging XAFS

**Figure 6:**
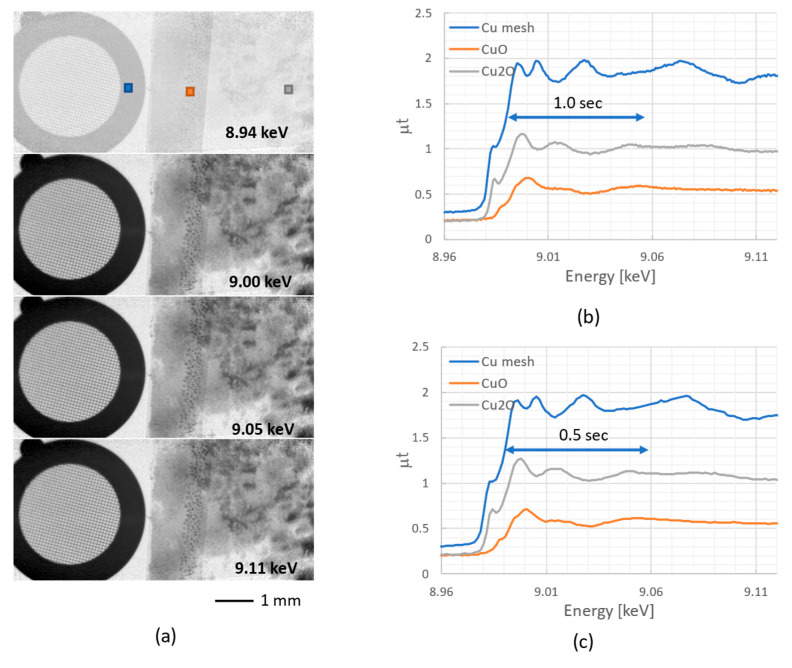
(**a**) Transmission images near the Cu K-edge (every 56 eV). (**b**) Absorption spectra in each region of Cu (blue), CuO (orange), and Cu_2_O (gray). (**c**) The same results obtained by SR energy scanning at twice the speed.

A feasibility test of quick imaging XAFS combined with high-speed SR energy scanning using a compact Ge DCM [42] was performed at BL07. A Cu 400 L/S mesh and Cu_2_O and CuO powder-sprinkled polyimide film (Cu_2_O particle size: 3 μm; CuO particle size: 5 μm) were used for a demonstrative sample. The ROI was set to 800 × 480 pixels, the exposure time was 10 ms, and the image transfer rate was 100 fps. The SR energy was continuously scanned by oscillating the main axis of the Ge DCM in a triangular (zigzag) pattern. The scan was centered at 12.1° (slightly above the Cu K-edge angle), with a range of ±0.1° (±1000 pulses) and a scanning speed of 0.1°/s (1000 pulses/s). During the scan, 2000 images were captured via manual triggering while monitoring the axis angle on the DCM controller. The time required for one way of the zigzag scan was 2.5 s (reduced to 1.3 s in Figure 6c), including the time for acceleration, deceleration, and controller overhead. Background images (*I*_BK_) were first acquired without the sample, followed by the acquisition of transmission images (*I*). Post-measurement, a single-scan image dataset was manually extracted from the 2000 acquired images: 250 frames for the 2.5 S scan (Figure 6b) and 125 frames for the 1.3 S scan (Figure 6c), by identifying the steep transmittance change at the Cu K-edge. The XAFS image data, which is the spatial distribution of the absorption μ*t* (μ: absorption coefficient, *t*: sample thickness) for each SR energy, was calculated by using the formula −ln(*I*/*I*_BK_) for each energy and pixel.

Figure 6a shows the obtained images taken at 75-frame intervals (8941.9, 8996.3, 9051.3, and 9107.0 eV). Absorption clearly increases sharply when the X-ray energy exceeds the Cu absorption K-edge (8.98 keV). Figure 6b shows the calculated μ*t* values, i.e., the absorption (XAFS) spectra, for each square region (10 × 10 pixels) indicated in Figure 6a: blue for the Cu mesh grid, orange for the CuO powder, and gray for the Cu_2_O powder. The blue line (Cu mesh) shows μ*t* variations (spectral shapes) similar to those of typical Cu foil. In contrast, the orange line (Cu_2_O powder) exhibits a shift in peak position, and the gray line (CuO powder) shows a greater pre-edge depth, indicating shapes representative of the chemical state of each sample [44]. These results show that the spatial distribution of the chemical state can be determined by using XAFS image data. It should be noted that in imaging XAFS, the X-ray energy generally shifts spatially because the incident angle to the DCM crystal varies slightly along the vertical direction. Although such energy shifts may occur in our measurements, no corrections were applied to the spectra shown in Figure 6b,c, as each of the square regions indicated in Figure 6a is located at approximately the same height. For future applications involving practical samples, we plan to implement energy calibration using a uniform Cu foil.

As the next step, measurements were performed on the same sample. The exposure time was the same (10 ms), and only the energy scan time was shortened. Figure 6c shows the XAFS spectra for each region obtained by scanning SR energy at 0.2 deg/s (2000 pls/s), which is twice the speed of (b). Shortening the scan time reduces the number of measurement points and decreases the energy resolution. However, the spectral shape is nearly identical to that in (b), except for a slight decrease in the pre-edge depth. Therefore, limiting the measurement to the X-ray Absorption Near Edge Structure (XANES) region allows for spectral acquisition in less than 0.5 s. This enables chemical state mapping to be performed with the same time resolution over time.

### 3.3. In Coquendo 4DCT

**Figure 7:**
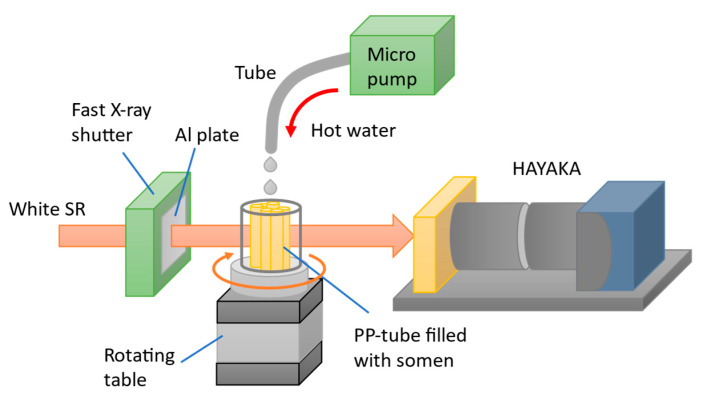
Schematic view of the *somen* boiling process observation system (*in coquendo* 4DCT observation system).

A feasibility test of a novel method, *in coquendo* 4DCT observation, which three-dimensionally non-destructively observes the cooking process over time using time-resolved CT, was performed by combining Hayaka with a high-speed rotating table. For *in coquendo* 4DCT observation of the noodles’ cooking process over time, we developed a system to observe the somen boiling process, as shown in Figure 7. Several short pieces of somen (Japanese noodles) were packed into a 6 mm-diameter polypropylene (PP) tube. While the tube itself was rotating, hot water was dripped into it from above, simulating the noodle boiling process. Note that the hot water drained out through a small hole opened at the bottom of the tube.

The observations were performed using white SR at BL07, as was performed in the previous section. A 0.5 mm-thick aluminum absorber was placed downstream of the TC1 slit. The table rotation speed was set to 2 revolutions per second, the ROI was 736 × 220 pixels, the camera exposure time was 9 μs, and the frame rate was set to 1000 fps. The filtered back-projection method (FBP) with the Shepp-Logan filter was used to reconstruct CT sectional images, with 500 projections per 360 degrees. Consequently, the time needed for a single CT scan, i.e., the temporal resolution, was 0.5 s. Hot water was delivered from a remotely controlled micropump through a tube to the top of the noodles and dripped onto them. The flow rate was 0.1 mL/s. The temperature of the hot water decreased as it traveled through the tube to 60 °C when it dripped onto the noodles. The measurement was started from the somen in a dry state, with dripping hot water added after 5 s, and continued for 150 s.

**Figure 8:**
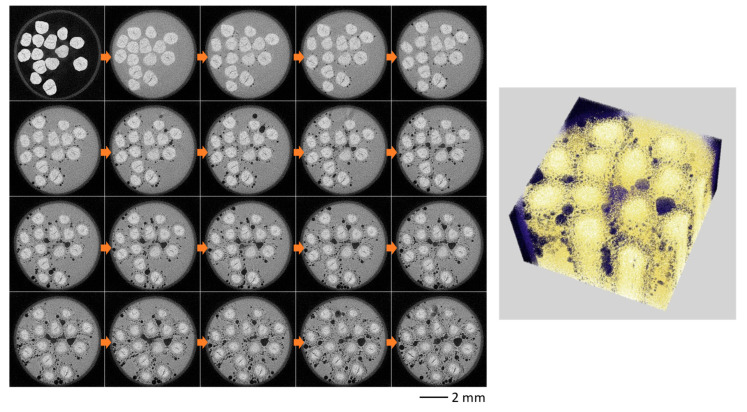
*In coquendo*, 4DCT observation results of hand-made somen. Sectional images every 5 s (**left**) and 3D volume rendering image at 75 s (**right**).

Figure 8 shows CT sectional and 3D volume rendering images of hand-made somen every five seconds (see Supplementary Materials for the 4D-CT movies S1 and S2). It is clear that hot water enters the tube, fine bubbles continuously form on the noodle surface, and the bubbles grow larger over time. At the same time, a crack that was present from the beginning gradually grows larger within the noodle’s core. Furthermore, as the hot water gradually penetrated the noodles, the density of the peripheral regions decreased, resulting in lower CT values. Consequently, both the cooked and uncooked regions could be clearly distinguished based on their respective CT values.

**Figure 9:**
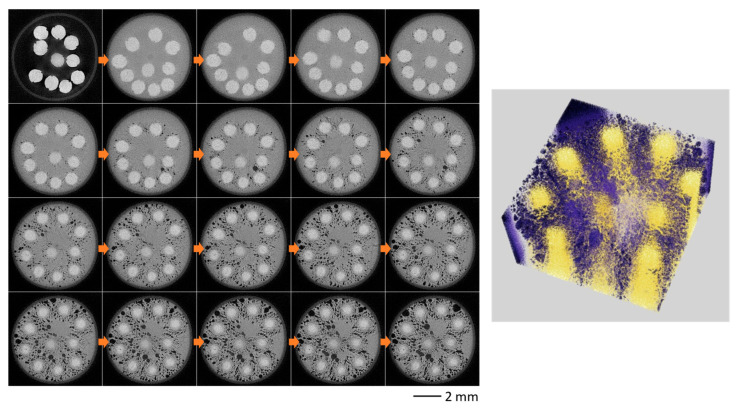
*In coquendo*, 4DCT observation results of machine-made somen. Sectional images every 5 s (**left**) and 3D volume rendering image at 75 s (**right**).

Figure 9 shows the results of observing machine-made somen under the same conditions (see Supplementary Materials for the 4D-CT movies S3 and S4). Similar to Figure 8, bubbles form and gradually grow; however, they are larger than the bubbles in hand-made somen. On the other hand, no cracks were present in the machine-made somen from the beginning, and the core (uncooked) region is smaller than that for hand-made somen. The supplement includes a series of time-resolved sectional images and 3D volume renderings with a time resolution of 0.5 s from the observations.

**Figure 10:**
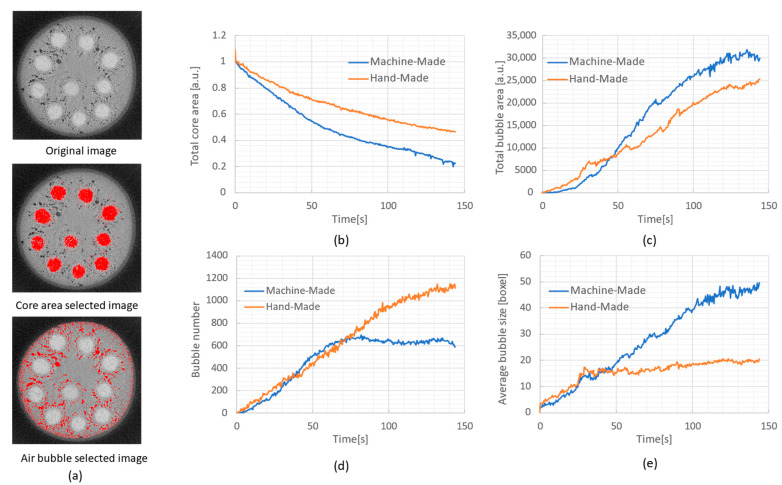
Results of quantitative analysis of CT sectional images. (**a**) Example images of original (**top**), extracted core area (**middle**), and bubbles (**bottom**). Temporal change in the (**b**) core area, (**c**) total bubble area, (**d**) bubble number, and (**e**) bubble size.

To demonstrate the powerful analytical capabilities of *in coquendo* 4DCT observation, we quantitatively analyzed the temporal changes in the core (uncooked) region, bubble area, bubble size, and bubble number using the obtained time-resolved CT sectional images in Figure 8 and Figure 9. First, we extracted the uncooked core area of somen and the bubbles by setting the threshold in Fiji [45], respectively, as shown in Figure 10a. Then, we performed a particle analysis on the extracted regions using “Analyze Particles” in Fiji. Figure 10 shows the temporal changes in the total core area (b), total bubble area (c), bubble number (d), and average bubble size (e). Figure 10b reveals that boiling progresses faster for machine-made somen than for hand-made somen, and the core area of the machine-made somen decreases from the initial value to approximately 20% within 150 s. While Figure 10c shows little difference in bubble area between the two types of somen, (d) and (e) reveal unexpected phenomena. Hand-made somen has smaller bubbles that continuously increase in number. In contrast, the number of bubbles in machine-made somen stops increasing around 60 s, followed by an increase in bubble size.

These results differ from those in our previous measurement [46] (which suggested hand-made somen boiled faster, etc.). The main reasons are considered to be (1) the low water temperature of 60 °C and (2) the low flow rate of the dripped hot water, which caused the bubbles to remain on the noodle surface, differing from a normal boiling state. We plan to attempt the measurement again in the future by creating an environment closer to actual boiling conditions, such as using hot water close to 100 °C and increasing the flow rate.

## 4. Conclusions

A lens-coupled high-speed X-ray camera, “Hayaka”, was developed for quick imaging of X-ray absorption fine structure (XAFS) and time-resolved high-speed computed tomography (CT) (4DCT) using synchrotron radiation (SR). Feasibility imaging using white SR at the beamline BL07 of SAGA Light Source succeeded in acquiring projection images with an exposure time of 10 μs and a spatial resolution of 77 μm. Quick imaging XAFS combined with high-speed energy scanning using a Ge double crystal monochromator (DCM) was tested, and spectral image data near the Cu K-edge region were successfully obtained in a minimum of 0.5 s. Furthermore, *in coquendo* 4DCT, which non-destructively observes cooking processes over time using time-resolved CT, was applied for the somen boiling process and successfully visualized the penetration of hot water into the noodle interior with a temporal resolution of 0.5 s.

In the next step, we are planning to attempt time-resolved chemical state mapping of various elements inside the electrodes during the battery charging/discharging relaxation process, after developing a synchronization system for Hayaka and the Ge DCM. Also, we are planning to develop an *in coquendo* 4DCT system that will enable observation of various cooking processes such as boiling, baking, steaming, frying, and heating with a microwave oven. Non-destructive and time-dependent clarification of these processes is expected to lead to the development of novel cooking methods and delicious recipes that reduce food loss.

## Figures and Tables

**Table 1:** Main specifications of EoSens 1.1CXP2.

Number of Pixels	1280 × 864	Frame Rate	3660 fps for Full Image 5000 fps for 800 × 400 Image
Pixel size	13.7 μm	Interface	CoaXPress 2.0 @ 12.5 Gbit/s
Exposure time	1 μs~1 s	Cooling	Air-cooling
Pixel data width	10 bits	Mount	C-mount

**Table 2:** Specifications of the high-NA lens used in the lens combination.

No.	Model No.	Focal Length	F Number	Supported Sensor Size	Mount
1	43F2409M-MP C	24 mm	0.9	4/3′′	C-Mount
2	43F2408M-MP	24 mm	0.8	4/3′′	M42 P1.0
3	VS-50085/C	50 mm	0.85	4/3′′	C-Mount

**Table 3:** Evaluation results of the effective width and height, required exposure time, and spatial resolution of lens combinations ① to ⑤.

No.	Pixel Size [μm]	Effective Width [mm]	Effective Height [mm]	Required Exposure Time for White SR [μs]	Required Exposure Time for Mono. SR [ms]	Spatial Resolution [μm]
①	13.7	4.5	2.0	15	36	70
②	13.7	5.4	2.3	12	32	77
③	26.5	11.9	2.7	11	30	90
④	6.8	6.3	2.6	40	88	55
⑤	6.8	6.9	2.8	32	68	70

## Data Availability

Dataset available on request from the authors.

## Supplementary Materials

The following supporting information can be downloaded at https://www.mdpi.com/article/10.3390/s26020434/s1. Video S1: video of the sectional images of hand-made somen during the boiling process. Video S2: video of the 3D volume rendering of hand-made somen during the boiling process. Video S3: video of the sectional images of machine-made somen during the boiling process. Video S4: video of the 3D volume rendering of machine-made somen during the boiling process.
